# Hypermultiplet gaugings and supersymmetric solutions from 11D and massive IIA supergravity on $\hbox {H}^{(p,q)}$ spaces

**DOI:** 10.1140/epjc/s10052-018-5672-9

**Published:** 2018-03-10

**Authors:** Adolfo Guarino

**Affiliations:** Service de Physique Théorique et Mathématique, Université Libre de Bruxelles (ULB) and International Solvay Institutes, Campus de la Plaine, CP 231, 1050 Brussels, Belgium

## Abstract

Supersymmetric $\text {AdS}_{4}$, $\text {AdS}_{2} \times \Sigma _{2}$ and asymptotically $\hbox {AdS}_{4}$ black hole solutions are studied in the context of non-minimal $\mathcal {N}=2$ supergravity models involving three vector multiplets (STU-model) and Abelian gaugings of the universal hypermultiplet moduli space. Such models correspond to consistent subsectors of the $\text {SO}(p,q)$ and $\text {ISO}(p,q)$ gauged maximal supergravities that arise from the reduction of 11D and massive IIA supergravity on $\text {H}^{(p,q)}$ spaces down to four dimensions. A unified description of all the models is provided in terms of a square-root prepotential and the gauging of a duality-hidden symmetry pair of the universal hypermultiplet. Some aspects of M-theory and massive IIA holography are mentioned in passing.

## Motivation

Asymptotically anti-de Sitter ($\hbox {AdS}_{4}$) black holes in minimal $\mathcal {N}=2$ gauged supergravity have recently been connected to a universal renormalisation group (RG) flow for a large class of three-dimensional $\mathcal {N}=2$ superconformal field theories (SCFTs) using holography [1]. The relevant (universal) $\hbox {AdS}_{4}$ black hole is static, extremal (thus $T=0$) and of Reissner–Nordström (R–N) type with zero mass and a hyperbolic horizon $\Sigma _{2}=\mathbb {H}^2$ [2]. The space-time metric takes the form

1 $$\begin{aligned} d s^2 = - e^{2U} d t^2 + e^{-2U} d r^2 + r^2 d\Omega _{\Sigma _2}, \end{aligned}$$

with

2 $$\begin{aligned} e^{2 U} = \left( \frac{r}{L_{\text {AdS}_{4}}} - \frac{L_{\text {AdS}_{4}}}{2 r} \right) ^2, \end{aligned}$$

and $d\Omega _{\Sigma _2}=d \theta ^2 + \sinh ^2(\theta ) d \phi ^2$ being the Riemann surface element on $\Sigma _{2}=\mathbb {H}^2$. The metric asymptotes an $\hbox {AdS}_{4}$ geometry with radius $L_{\text {AdS}_{4}}$ when ${r \rightarrow \infty }$, and conforms to ${\text {AdS}_{2} \times \mathbb {H}^2}$ in the near-horizon region $r \rightarrow r_{h}=L_{\text {AdS}_{4}}/\sqrt{2}$ after a shift of the radial coordinate $r \rightarrow r + r_{h}$ and the identification $L_{\text {AdS}_{2}}^2 = \tfrac{1}{4} L_{\text {AdS}_{4}}^2$. This black hole is a solution of the equations of motion that follow from the cosmological Einstein–Maxwell Lagrangian

3 $$\begin{aligned} \mathcal {L} = \left( \tfrac{1}{2} R - V \right) * 1 - \tfrac{1}{2} \mathcal {H} \wedge * \mathcal {H}. \end{aligned}$$

Endowing the Lagrangian (3) with $\mathcal {N}=2$ local supersymmetry requires the cosmological constant to be negative and also a mass term for the gravitini fields in the theory [3]. Furthermore, supersymmetry fixes the cosmological constant to ${V=-3 L_{\text {AdS}_{4}}^{-2}=-3 |\mu |^2}$ in terms of the mass $\mu$ of a (single) complex gravitino, and renders the black hole magnetically charged [2] (see also [4]), i.e. ${\mathcal {H} = p \sinh (\theta ) d\theta \wedge d\phi }$, with the constant flux *p* being also set by supersymmetry.

The $\hbox {AdS}_{4}$ black hole described above is gauge/gravity dual to a universal RG flow in field theory [1]. This is an RG flow across dimensions^1^ from a three-dimensional $\mathcal {N}=2$ SCFT (dual to the asymptotic $\hbox {AdS}_{4}$ geometry) placed on $\mathbb {H}^{2}$ and with a topological twist along the exact superconformal R-symmetry, to a one-dimensional superconformal quantum mechanics (dual to the $\hbox {AdS}_{2}$ factor of the black hole near-horizon geometry). Such a universal RG flow admits various holographic embeddings in eleven-dimensional (11D) supergravity [6, 7], the low-energy limit of M-theory, and ten-dimensional massive IIA supergravity [8]. Specific examples have been studied in the context of ABJM theory [9] and GJV/SYM-CS duality [10] (and its generalisation of [11]) involving reductions of M-theory and massive IIA strings on various compact spaces [1]. More concretely, when placing the SCFTs on $\text {S}^1 \times \Sigma _{2}$, a counting of supersymmetric ground states using the topologically twisted index of [12] at large *N* was shown to exactly reproduce the Bekenstein–Hawking entropy associated with the $\hbox {AdS}_4$ black hole in (1), (2).

Extensions to non-minimal $\mathcal {N}=2$ supergravity coupled to matter multiplets, namely, $n_{v}$ vector multiplets and $n_{h}$ hypermultiplets, have also been investigated in the context of M-theory [13–15] and massive IIA strings [16, 17]. In the M-theory case, the relevant gauged supergravity is the so-called STU-model with $(n_{v},n_{h})=(3,0)$ and Fayet-Iliopoulos gaugings with equal gauging parameters $g_{\Lambda }=g$. This model arises from 11D supergravity when reduced on a compact seven-sphere $\text {H}^{(8,0)}\equiv \text {S}^7$ [18] down to a four-dimensional SO(8) gauged maximal supergravity [19], and further truncated to its $\text {U}(1)^4$ invariant subsector [20, 21]. The gauging parameter *g* is then identified with the inverse radius of $\text {S}^7$. In the massive IIA case, the relevant gauged supergravity has $(n_{v},n_{h})=(3,1)$ and involves Abelian gaugings of the universal hypermultiplet moduli space with gauging parameters *g* and *m*. This model arises from ten-dimensional massive IIA supergravity when reduced on a compact six-sphere $\text {H}^{(7,0)}\equiv \text {S}^6$ [22] down to a four-dimensional ISO(7) gauged maximal supergravity [23, 24] of dyonic type [25], and further truncated to its $\text {U}(1)^2$ invariant subsector [26]. The gauging parameter *g* is again identified with the inverse radius of $\text {S}^6$ whereas $\,m\,$ corresponds to the Romans mass parameter. Supersymmetric $\hbox {AdS}_{4}$ black holes generalising the one in (1), (2) have been constructed in such M-theory [27] and massive IIA [26, 28] non-minimal $\,\mathcal {N}=2\,$ supergravity models.

In this note we build upon the above results and study non-minimal $\,\mathcal {N}=2\,$ supergravity models that arise from 11D and massive IIA supergravity when reduced on $\,\text {H}^{(p,q)}=\text {SO}(p,q)/\text {SO}(p-1,q)\,$ homogeneous spaces with various $\,(p,q)\,$ signatures. In the case of 11D supergravity, the zero-mass sector recovers an electrically-gauged maximal supergravity in four dimensions with $\,\text {SO}(p,q)\,$ gauge group and $\,{p+q=8}\,$ [29]. In the case of ten-dimensional massive IIA supergravity, the reduction on $\,\text {H}^{(p,q)}\,$ spaces down to four dimensions yields a dyonically-gauged maximal supergravity with $\,\text {ISO}(p,q)=\text {CSO}(p,q,1)\,$ gauge group and $\,p+q=7\,$ [30–32]. We provide a systematic characterisation and a unified description of the $\,\text {U}(1)^2\,$ invariant sectors associated with such M-theory and massive IIA reductions.^2^ The resulting models describe non-minimal $\,\mathcal {N}=2\,$ supergravity with $\,(n_{v},n_{h})=(3,1)\,$ and involve Abelian gaugings of the universal hypermultiplet moduli space. Supersymmetric $\,\text {AdS}_{4}\,$, $\,\text {AdS}_{2} \times \Sigma _{2}\,$ and universal $\,\text {AdS}_{4}\,$ black hole solutions are systematically studied which, upon uplifting to eleven- or ten-dimensional backgrounds, are of interest for M-theory and massive IIA holography.

## $\mathcal {N}=2$ supergravity models

The bosonic sector of $\,\mathcal {N}=2\,$ supergravity coupled to $\,n_{v}\,$ vector multiplets and $\,n_{h}\,$ hypermultiplets is described by a Lagrangian of the form [33]

4 $$\begin{aligned} \mathcal {L}= & {} \left( \frac{R}{2} - V \right) * 1 - K_{i\bar{j}} \, Dz^{i} \wedge * D\bar{z}^{\bar{j}} - h_{uv} \, Dq^{u} \wedge * Dq^{v} \nonumber \\&+ \frac{1}{2} \, \mathcal {I}_{\Lambda \Sigma } \, \mathcal {H}^\Lambda \wedge * \mathcal {H}^\Sigma + \frac{1}{2} \, \mathcal {R}_{\Lambda \Sigma } \, \mathcal {H}^\Lambda \wedge \mathcal {H}^\Sigma \nonumber \\&+ \frac{1}{2} \Theta ^{\Lambda \alpha }\, \mathcal {B}_{\alpha } \wedge d \tilde{\mathcal {A}}_{\Lambda } + \frac{1}{8} \, \Theta ^{\Lambda \alpha } \, \Theta _{\Lambda }{}^{\beta } \, \mathcal {B}_{\alpha } \wedge \mathcal {B}_{\beta }. \end{aligned}$$

In this note we focus on models with three vector multiplets and the universal hypermultiplet [34], namely, $\,(n_{v}, n_{h})=(3,1)$.

The three complex scalars $\,{z^{i}=-\chi _{i}+ i \, e^{-\varphi _{i}}}\,$ in the vector multiplets $(i=1,2,3)$ serve as coordinates in the special Kähler (SK) manifold $\,\mathcal {M}_{\text {SK}}=[\text {SU}(1,1)/\text {U}(1)]^{3}$. The metric on this manifold is given by

5 $$\begin{aligned} ds_{\text {SK}}^2 = K_{i\bar{j}} \, dz^{i} d\bar{z}^{\bar{j}} = \frac{1}{4} \sum _{i}\frac{dz^{i} \, d\bar{z}^{\bar{i}}}{(\text {Im} z^{i})^2}, \end{aligned}$$

where $\,K_{i \bar{j}} = \partial _{z^{i}} \partial _{\bar{z}^{\bar{j}}} K\,$ and $\,{K=-\log (i \left\langle X, \bar{X} \right\rangle )}\,$ is the real Kähler potential. The latter is expressed in terms of an $\,\text {Sp}(8)\,$ symplectic product $\,\left\langle X, \bar{X} \right\rangle =X^{M} \Omega _{MN} \bar{X}^{N}=X_{\Lambda }\bar{X}^{\Lambda }-X^{\Lambda }\bar{X}_{\Lambda }\,$ of holomorphic sections $\,X^{M}(z^{i})=(X^{\Lambda },F_{\Lambda })\,$ that satisfy $\,F_{\Lambda } = \partial \mathcal {F}/\partial X^{\Lambda }\,$ ($\Lambda =0,1, 2, 3$) for a homogeneous prepotential of degree-two $\,\mathcal {F}(X^{\Lambda })$. Our choice of sections

6 $$\begin{aligned} X^{M} = (-z^{1} z^{2} z^{3},-z^{1},-z^{2},-z^{3},1, z^{2} z^{3}, z^{3} z^{1}, z^{1} z^{2}), \end{aligned}$$

is compatible with a square-root prepotential

7 $$\begin{aligned} \mathcal {F} = -2 \sqrt{X^0 \, X^1 \, X^2 \, X^3}, \end{aligned}$$

and restricts the range of the $\,z^{i}\,$ scalars to the Kähler cone

8 $$\begin{aligned} i \left\langle X, \bar{X} \right\rangle = 8 \, \text {Im} z^{1} \, \text {Im} z^{2} \, \text {Im} z^{3} > 0. \end{aligned}$$

The condition in (8) leaves two different domains: either the three $\,\text {Im} z^{i}\,$ are positive, or two of them are negative and the third one is positive.

The kinetic terms and the generalised theta angles for the vector fields in (4) are given by $\,\mathcal {R}_{\Lambda \Sigma }=\text {Re}(\mathcal {N}_{\Lambda \Sigma })\,$ and $\,\mathcal {I}_{\Lambda \Sigma }=\text {Im}(\mathcal {N}_{\Lambda \Sigma })\,$ in terms of a complex matrix

9 $$\begin{aligned} \mathcal {N}_{\Lambda \Sigma } = \bar{F}_{\Lambda \Sigma }+ 2 i \frac{\text {Im}(F_{\Lambda \Gamma })X^{\Gamma }\,\,\text {Im}(F_{\Sigma \Delta })X^{\Delta }}{\text {Im}(F_{\Omega \Phi })X^{\Omega }X^{\Phi }}, \end{aligned}$$

with $\,F_{\Lambda \Sigma }=\partial _{\Lambda }\partial _{\Sigma } \mathcal {F}$. They can be used to define a symmetric, real and negative-definite scalar matrix

10 $$\begin{aligned} \mathcal {M}(z^{i}) = \left( \begin{array}{cc} \mathcal {I} + \mathcal {R} \mathcal {I}^{-1} \mathcal {R} &{} -\mathcal {R} \mathcal {I}^{-1} \\ - \mathcal {I}^{-1} \mathcal {R} &{} \mathcal {I}^{-1} \end{array} \right) , \end{aligned}$$

that will appear later on in Sec. 4.2. The vector field strengths are given by

11 $$\begin{aligned} \mathcal {H}^{\Lambda } = d \mathcal {A}^{\Lambda } - \tfrac{1}{2} \, \Theta ^{\Lambda \alpha } \, \mathcal {B}_{\alpha }, \end{aligned}$$

and incorporate a set of tensor fields $\,\mathcal {B}_{\alpha }\,$ where $\,\alpha \,$ is a collective index running over the isometries of the scalar manifold that are gauged. Importantly, the tensor fields enter the Lagrangian in (4) provided that the couplings $\,\Theta ^{\Lambda \alpha } \ne 0\,$, namely, if magnetic charges (see Eq. (13) below) are present in the theory [33]. Tensor fields will play a role in the case of massive IIA reductions on $\,\text {H}^{(p,q)}\,$ spaces as magnetic charges are induced by the Romans mass parameter.

The quaternionic Kähler (QK) manifold spanned by the four real scalars $\,q^{u}=(\,\phi \,,\sigma \,,\,\zeta \,,\,\tilde{\zeta }\,)\,$ in the universal hypermultiplet is $\,{\mathcal {M}_{\text {QK}}=\text {SU}(2,1)/\text {SU}(2)\times \text {U}(1)}$. The metric on this QK space reads

12 $$\begin{aligned} ds_{\text {QK}}^2= & {} d \phi \, d \phi + \frac{1}{4} e^{4 \phi } \left[ d \sigma + \tfrac{1}{2} \, \mathbf {\zeta } \, \mathbb {C} \, d \mathbf {\zeta } \right] \, \left[ d \sigma + \tfrac{1}{2} \, \mathbf {\zeta } \, \mathbb {C} \, d \mathbf {\zeta } \right] \nonumber \\&+ \frac{1}{4} \, e^{2\phi } \, d \mathbf {\zeta } \, d\mathbf {\zeta }, \end{aligned}$$

with $$\,\mathbb {C}=\left( \begin{array}{cc} 0 &{} 1 \\ - 1 &{} 0\end{array} \right) \,$$ and $\,\mathbf {\zeta }=(\zeta ,\tilde{\zeta })$. In this note we are exclusively interested in gauging Abelian isometries of $\,{\mathcal {M}_{\text {QK}}}\,$ as dictated by an embedding tensor $\,\Theta _{M}{}^{\alpha }\,$ [33]. They have associated Killing vectors $\,k^{u}_{\alpha }\,$ and yield covariant derivatives in (4) of the form

13 $$\begin{aligned} Dz^{i} = d z^{i} \quad \text { and } \quad D q^{u} = d q^{u} - \mathcal {A}^{M} \, \Theta _{M}{}^{\alpha } \, k_{\alpha }^{u}. \end{aligned}$$

Lastly it turns also convenient to introduce symplectic Killing vectors $\,{\mathcal {K}_{M} \equiv \Theta _{M}{}^{\alpha } \, k_{\alpha }}\,$ and moment maps $\,\mathcal {P}_{M}^{x} \equiv \Theta _{M}{}^{\alpha } \, P_{\alpha }^{x}\,$ in order to maintain symplectic covariance [35]. The scalar potential in (4) can then be expressed as [33, 36]

14 $$\begin{aligned} V= & {} 4 \, \mathcal {V}^{M} \, \bar{\mathcal {V}}^{N} \, \mathcal {K}_{M}{}^{u} \, h_{uv} \, \mathcal {K}_{N}{}^{v} \nonumber \\&+\, \mathcal {P}^{x}_{M} \, \mathcal {P}^{x}_{N} \left( K^{i\bar{j}} \, D_{i}\mathcal {V}^{M} \, D_{\bar{j}} \bar{\mathcal {V}}^{N} - 3 \, \mathcal {V}^{M} \, \bar{\mathcal {V}}^{N} \right) , \end{aligned}$$

in terms of rescaled sections $\,\mathcal {V}^{M} \equiv e^{K/2} \, X^{M}\,$ and their Kähler derivatives $\,D_{i}\mathcal {V}^{M}=\partial _{z^{i}} \mathcal {V}^{M} + \frac{1}{2} (\partial _{z^{i}} K)\mathcal {V}^{M}$.

## Abelian hypermultiplet gaugings from reductions on $\text {H}^{(p,q)}$

The metric (12) on the special QK manifold associated with the universal hypermultiplet lies in the image of a c-map [37–39] with a trivial special Kähler base. There are three isometries $\,k_{\alpha }=\{ k_{\sigma }, \widehat{k}_{\sigma }, \, k_{\mathbb {U}} \}\,$ of (12) that play a role in our reductions of 11D and massive IIA supergravity on $\,\text {H}^{(p,q)}\,$ spaces. The isometry $\, k_{\sigma } \,$ corresponds to a model-independent (axion shift) duality symmetry. On the contrary, the isometry $\, \widehat{k}_{\sigma }\,$ corresponds to a hidden symmetry. Together, they form a duality-hidden symmetry pair (conjugate roots of the global symmetry algebra) given by

15 $$\begin{aligned} k_{\sigma }= & {} -\partial _{\sigma }, \nonumber \\ \widehat{k}_{\sigma }= & {} \sigma \, \partial _{\phi } - (\sigma ^2 - e^{-4 \phi } - U) \, \partial _{\sigma } \nonumber \\&- \left[ \, \sigma \mathbf {\zeta } - \mathbb {C} \, (\partial _{\mathbf {\zeta }} U) \, \right] ^{T} \, \partial _{\mathbf {\zeta }}, \end{aligned}$$

with

16 $$\begin{aligned} U = \frac{1}{16} \, |\mathbf {\zeta }|^4 \, + \, \frac{1}{2} \, e^{-2 \phi } \, |\mathbf {\zeta }|^2. \end{aligned}$$

The remaining isometry $\,k_{\mathbb {U}}\,$ corresponds to a model-dependent duality symmetry and, for the M-theory and massive IIA models studied in this note, it is given by

17 $$\begin{aligned} k_{\mathbb {U}}= & {} \tilde{\zeta } \, \partial _{\zeta } - \zeta \, \partial _{\tilde{\zeta }}. \end{aligned}$$

Note that, while $\,\widehat{k}_{\sigma } + k_{\sigma }\,$ and $\,k_{\mathbb {U}}\,$ are compact Abelian isometries of (12), the combination $\,\widehat{k}_{\sigma } - k_{\sigma }\,$ turns to be non-compact.

The triplet of moment maps $\,P_{\alpha }^{x}\,$ associated to the Killing vectors in (15) and (17) can be obtained from the general construction of [40]. The Killing vectors in (15) have associated moment maps of the form

18 $$\begin{aligned} P^{x}_{\sigma } = \left( \begin{array}{c} 0 \\ 0 \\ -\frac{1}{2} \, e^{2 \phi } \end{array} \right) , \end{aligned}$$

and

19 $$\begin{aligned} \widehat{P}^{x}_{\sigma }&= \left( \begin{array}{c} - e^{-\phi } \, \tilde{\zeta } + e^{\phi }\, \left( \, - \sigma \, \zeta +\frac{1}{4} \, |\mathbf {\zeta }|^2 \,\tilde{\zeta } \, \right) \\ e^{-\phi } \, \zeta + e^{\phi }\, \left( \, - \sigma \, \tilde{\zeta } - \frac{1}{4} \, |\mathbf {\zeta }|^2 \, \zeta \, \right) \\ -\frac{1}{2} \, e^{-2 \phi } -\frac{1}{2} \, e^{2 \phi } \, \sigma ^2 -\frac{1}{32} \, e^{2 \phi } \, |\mathbf {\zeta }|^4 + \frac{3}{4} \, |\mathbf {\zeta }|^2 \end{array} \right) , \end{aligned}$$

whereas the moment maps for the isometry in (17) take the simpler form

20 $$\begin{aligned} P^{x}_{\mathbb {U}}= & {} \left( \begin{array}{c} e^{\phi } \, \tilde{\zeta } \\ - e^{\phi } \, \zeta \\ 1 - \frac{1}{4} \, e^{2 \phi } \, |\mathbf {\zeta }|^2 \end{array} \right) . \end{aligned}$$

**Table 1:** Embedding tensor (21) and gauged isometries. For the sake of definiteness, and without loss of generality, we are taking $\,p \ge q\,$ as well as $\,g>0\,$ and $\,m>0\,$

Gauging	$g_{0}$	$g_{1}$	$g_{2}$	$g_{3}$	$m_{0}$	$\,\,m_{i}\,\,$	$k_{1}$	$k_{2}$
$\text {SO}(8)$	*g*	*g*	*g*	*g*	0	0	$\,\,\widehat{k}_{\sigma } + k_{\sigma } \,\,$	$\,\,k_{\mathbb {U}}\,\,$
$\text {SO}(7,1)$	$-g$	*g*	*g*	*g*	0	0	$\,\,\widehat{k}_{\sigma } - k_{\sigma } \,\,$	$\,\,k_{\mathbb {U}}\,\,$
$\text {SO}(6,2)_{a}$	$-g$	*g*	*g*	*g*	0	0	$\,\,\widehat{k}_{\sigma } + k_{\sigma } \,\,$	$\,\,k_{\mathbb {U}}\,\,$
$\text {SO}(6,2)_{b}$	*g*	*g*	*g*	$-g$	0	0	$\,\,\widehat{k}_{\sigma } + k_{\sigma } \,\,$	$\,\,k_{\mathbb {U}}\,\,$
$\text {SO}(5,3)$	$-g$	*g*	*g*	$-g$	0	0	$\,\,\widehat{k}_{\sigma } - k_{\sigma } \,\,$	$\,\,k_{\mathbb {U}}\,\,$
$\text {SO}(4,4)$	$-g$	*g*	*g*	$-g$	0	0	$\,\,\widehat{k}_{\sigma } + k_{\sigma } \,\,$	$\,\,k_{\mathbb {U}}\,\,$
$\text {ISO}(7)$	*g*	*g*	*g*	*g*	*m*	0	$\,\,k_{\sigma } \,\,$	$\,\,k_{\mathbb {U}}\,\,$
$\text {ISO}(6,1)$	$-g$	*g*	*g*	*g*	*m*	0	$\,\,k_{\sigma } \,\,$	$\,\,k_{\mathbb {U}}\,\,$
$\text {ISO}(5,2)$	*g*	*g*	*g*	$-g$	*m*	0	$\,\,k_{\sigma } \,\,$	$\,\,k_{\mathbb {U}}\,\,$
$\text {ISO}(4,3)$	$-g$	*g*	*g*	$-g$	*m*	0	$\,\,k_{\sigma } \,\,$	$\,\,k_{\mathbb {U}}\,\,$

In order to fully determine the $\,\mathcal {N}=2\,$ supergravity model, one must still specify the gauge connection entering the covariant derivatives in (13). This is done by an embedding tensor $\,\Theta _{M}{}^{\alpha }\,$ of the form

21 $$\begin{aligned} \Theta _{M}{}^{\alpha } = \left( \begin{array}{c} \Theta _{\Lambda }{}^{\alpha } \\ \Theta ^{\Lambda \, \alpha } \end{array}\right) = \left( \begin{array}{cc} g_{0} &{} 0 \\ 0 &{} g_{1} \\ 0 &{} g_{2} \\ 0 &{} g_{3} \\ m_{0} &{} 0 \\ 0 &{} m_{1} \\ 0 &{} m_{2} \\ 0 &{} m_{3} \end{array}\right) , \end{aligned}$$

where the various electric $\,g_{\Lambda }\,$ and magnetic $\ m_{\Lambda }\,$ charges are displayed in Table 1. The covariant derivatives in (13) reduce to

22 $$\begin{aligned} D z^{i}= & {} d z^{i}, \nonumber \\ D q^{u}= & {} d q^{u} - (g_{0} \, \mathcal {A}^{0} + m_{0} \, \mathcal {\tilde{A}}_{0}) \, k_{1}^{u} - \mathcal {A} \, k_{2}^{u} \, \end{aligned}$$

with $\,\mathcal {A}=\sum _{i} g_{i} \, \mathcal {A}^{i} + \sum _{i} m_{i} \, \tilde{\mathcal {A}}_{i}$. However, the specific isometries $\,k_{1}\,$ and $\,k_{2}\,$ to be gauged in (22) depend on the M-theory or massive IIA origin of the models:

23 $$\begin{aligned} \begin{array}{lll} \text {M-theory} : &{} k_{1} =\widehat{k}_{\sigma } + (-1)^{p q} \, k_{\sigma } &{} k_{2}=k_{\mathbb {U}} , \\ \text {Massive IIA} : &{} k_{1} = k_{\sigma } , &{} k_{2}= k_{\mathbb {U}}. \\ \end{array} \end{aligned}$$

The $\,(-1)^{p q}\,$ sign in $\,k_{1}\,$ for the M-theory models depends on the signature of the $\,\text {H}^{(p,q)}\,$ space employed in the reduction. Moreover, as a consequence of (23), only duality symmetries $\,k_{\sigma }\,$ and $\,k_{\mathbb {U}}\,$ appear upon reductions of massive IIA supergravity whereas also the hidden symmetry $\,\widehat{k}_{\sigma }\,$ does it in the reductions of M-theory. Note also that, in the massive IIA case, the resulting gaugings are of dyonic type: they involve both electric and magnetic charges.

## Supersymmetric solutions

The M-theory and massive IIA non-minimal $\,{\mathcal {N}=2}\,$ supergravity models presented in the previous section possess various types of supersymmetric solutions.

### $\text {AdS}_{4}$ solutions

An $\,\text {AdS}_{4}\,$ vacuum solution with radius $\,L_{\text {AdS}_{4}}\,$ is describing a space-time geometry of the form

24 $$\begin{aligned} d s^2 = - \frac{r^2}{L^2_{\text {AdS}_{4}}} \, d t^2 + \frac{L^2_{\text {AdS}_{4}}}{r^2} \, d r^2 + r^{2} \, d\Omega _{\Sigma _2}, \end{aligned}$$

where $\,d\Omega _{\Sigma _2}=d \theta ^2 + \left( \frac{ \sin \sqrt{\kappa } \, \theta }{ \sqrt{ \kappa } } \right) ^2 \, d \phi ^2\,$ is the surface element of $\,\Sigma _{2}=\left\{ \mathbb {S}^2 \, (\kappa =+1), \, \mathbb {H}^2 \, (\kappa =-1) \right\}$. Being a maximally symmetric solution, only scalars are allowed to acquire a non-trivial and constant vacuum expectation value that extremises the potential in (14). In addition, preserving $\,{\mathcal {N}=2}\,$ supersymmetry requires the vanishing of all fermionic supersymmetry variations. This translates into the conditions [41]:

25 $$\begin{aligned} X^{M}\, \mathcal {K}_{M}= & {} 0 , \nonumber \\ \left[ \, \partial _{z^{i}} X^{M} + (\partial _{z^{i}} K) X^{M} \, \right] \, \mathcal {P}^{x}_{M}= & {} 0 , \nonumber \\ S_{\mathcal {AB}} \, \epsilon ^{\mathcal {B}}= & {} \frac{1}{2} \, \mu \, \epsilon ^{*}_{\mathcal {A}} , \end{aligned}$$

where $\,S_{\mathcal {AB}}=\frac{1}{2} e^{K/2} X^{M} \mathcal {P}^{x}_{M} (\sigma ^{x})_{\mathcal {AB}}\,$ is the gravitino mass matrix, $\,(\sigma ^{x})_{\mathcal {AB}}\,$ are the Pauli matrices and $\,|\mu |=L^{-1}_{\text {AdS}_{4}}$.

#### M-theory

In the $\,\text {SO}(p,q)\,$ theories with $\,k_{1}=\widehat{k}_{\sigma } + (-1)^{pq} \, k_{\sigma }\,$ the first condition in (25) yields

26 $$\begin{aligned} \sigma = 0 \text { and } \left( e^{-2 \phi } + \frac{1}{4} \, |\mathbf {\zeta }|^2\right) ^2 = (-1)^{pq}. \end{aligned}$$

The last equation in (26) excludes theories with $\,{p \, q \in \text {odd}}$, namely, the SO(7,1) and SO(5,3) theories. For those theories with $\,{p \, q \in \text {even}}\,$, the first condition in (25) additionally gives

27 $$\begin{aligned} \left( \sum _{i} g_{i} \, z^{i} + g_{0} \, \prod _{i} z^{i} \right) \, \mathbf {\zeta } = 0. \end{aligned}$$

Extremising the scalar potential in (14) subject to the conditions in (25) imposed by supersymmetry yields two $\hbox {AdS}_{4}$ solutions that preserve a different residual (unbroken) gauge symmetry.

The first solution fixes the scalars $\,z^{i}\,$ in the vector multiplets as

28 $$\begin{aligned} z^{i}= \pm \, i \, \dfrac{(g_{0} \, g_{1} \, g_{2} \, g_{3})^{\frac{1}{2}}}{g_{0} \, g_{i}}, \end{aligned}$$

and involves a trivial configuration of the scalars in the universal hypermultiplet

29 $$\begin{aligned} e^{\phi }= 1 \quad \text { and } \quad |\mathbf {\zeta }|^2=0. \end{aligned}$$

This solution possesses a realisation in two theories:

30 $$\begin{aligned}&\text {SO(8)} : z^{1}=z^{2}=z^{3}= i, \end{aligned}$$

31 $$\begin{aligned}&\text {SO(4,4)} : z^{1}=z^{2}=-z^{3}= -i. \end{aligned}$$

In both cases

32 $$\begin{aligned} L^2_{\text {AdS}_{4}} = \frac{1}{2 \, g^2}, \end{aligned}$$

and the two vectors $\,g_{0} \, \mathcal {A}^{0}\,$ and $\,\mathcal {A}\,$ remain massless ($k_{1}=k_{2}=0$), thus preserving the full $\,\text {U}(1)_{1} \times \text {U}(1)_{2}\,$ gauge symmetry of the models. In the compact $\,\text {SO}(8)\,$ case, this solution uplifts to the maximally supersymmetric Freund-Rubin vacuum of 11D supergravity [42], and the dual SCFT is identified with ABJM theory [9] at low ($\,k=1,2\,$) Chern–Simons levels $\,k\,$ and $\,-k$.

The second solution fixes the scalars $\,z^{i}\,$ at the values

33 $$\begin{aligned} z^{i}= \pm \, i \, \sqrt{3} \, \dfrac{(g_{0} \, g_{1} \, g_{2} \, g_{3})^{\frac{1}{2}}}{g_{0} \, g_{i}}, \end{aligned}$$

and involves a non-trivial configuration of the universal hypermultiplet independent of the gauging parameters

34 $$\begin{aligned} e^{\phi }= \dfrac{\sqrt{3}}{\sqrt{2}} \quad \text { and } \quad |\mathbf {\zeta }|^2=\frac{4}{3}. \end{aligned}$$

This solution has a realisation in the same theories as before:

35 $$\begin{aligned}&\text {SO(8)} : z^{1}=z^{2}=z^{3}= i \, \sqrt{3}, \end{aligned}$$

36 $$\begin{aligned}&\text {SO(4,4)} : z^{1}=z^{2}=-z^{3}= -i \, \sqrt{3}. \end{aligned}$$

In both cases

37 $$\begin{aligned} L^2_{\text {AdS}_{4}} = \frac{2}{3 \sqrt{3} \, g^2}, \end{aligned}$$

and only the linear combination of vectors $\,- \, 3 \, g_0 \, \mathcal {A}^{0} + \mathcal {A}\,$ remains massless ($k_{1}=k_{2}$) in the $\,\text {SO}(8)\,$ and $\,\text {SO}(4,4)\,$ theories. The associated $\,\text {U}(1)\,$ gauge symmetry is thus preserved and to be identified with the R-symmetry of the dual field theory. In the compact $\,\text {SO}(8)\,$ case, this solution was studied in [43, 44] and uplifted to a background of 11D supergravity in [45, 46]. Its dual SCFT was identified in [47] as the infrared fixed point of an RG flow from ABJM theory triggered by an SU(3) invariant mass term in the superpotential.

The fixing of the scalars $\,z^{i}\,$ in the vector multiplets to the values in (30), (31) and (35), (36) is compatible with the Kähler cone condition (8) for a physically acceptable solution in four dimensions.

#### Massive IIA

In the $\,\text {ISO}(p,q)\,$ theories the first condition in (25) yields

38 $$\begin{aligned} \left( \sum _{i} g_{i} \, z^{i} \right) \, \mathbf {\zeta } = 0 \quad \text { and } \quad \prod _{i} z^{i} = \frac{m_{0}}{g_{0}}. \end{aligned}$$

The extremisation of the scalar potential in (14) subject to the supersymmetry conditions in (25) yields this time a unique $\hbox {AdS}_{4}$ solution. It has

39 $$\begin{aligned} \chi _{i}^3 = \frac{1}{8} \, \dfrac{g_1 \, g_2 \, g_3}{g_{i}^3} \, \frac{m_{0}}{g_{0}},\quad e^{-3\varphi _{i}} = \pm \frac{3 \sqrt{3}}{8} \, \dfrac{g_1 \, g_2 \, g_3}{g_{i}^3} \, \frac{m_{0}}{g_{0}}, \end{aligned}$$

for the $\,z^{i}\,$ scalars in the vector multiplets, together with $\, |\mathbf {\zeta }|^2=0 \,$ and a non-trivial dilaton

40 $$\begin{aligned} e^{6 \phi }= 8 \,\, \dfrac{g_1 \, g_2 \, g_3}{g_0 \, m_{0}^2}, \end{aligned}$$

in the universal hypermultiplet. Note that (40) requires $\,g_0 \, g_1 \, g_2 \, g_3 > 0\,$ which is satisfied only by the ISO(7) and ISO(4,3) theories (see Table 1). The corresponding solutions are given by

41 $$\begin{aligned}&\text {ISO(7)} : z^{1}=z^{2}=z^{3}= \left( \tfrac{m}{g}\right) ^{\frac{1}{3}} \, e^{i \frac{2\pi }{3}}, \end{aligned}$$

42 $$\begin{aligned}&\text {ISO(4,3)} : z^{1}=z^{2}=-z^{3}= \left( \tfrac{m}{g}\right) ^{\frac{1}{3}} e^{-i \frac{2\pi }{3}}, \end{aligned}$$

together with

43 $$\begin{aligned} e^{\phi }= \sqrt{2} \, \left( \frac{g}{m}\right) ^{\frac{1}{3}}. \end{aligned}$$

Both configurations (41), (42) satisfy the Kähler cone condition (8) and yield

44 $$\begin{aligned} L^2_{\text {AdS}_{4}} = \tfrac{1}{\sqrt{3}} \, g^{-\frac{7}{3}}\, m^{\frac{1}{3}}. \end{aligned}$$

The vector $\,\mathcal {A}\,$ remains massless ($k_{2}=0$) and so the $\,\text {U}(1)_{2}\,$ gauge symmetry is preserved. This symmetry is holographically identified with the R-symmetry of the dual field theory. In the ISO(7) case, this solution was presented in [10, 24]^3^ and uplifted to a background of massive IIA supergravity in [10, 22]. The dual three-dimensional SCFT was also identified in [10] as a super Chern–Simons-matter theory with simple gauge group $\text {SU}(N)$ and level $\,k\,$ given by the Romans mass parameter.

### $\text {AdS}_{2} \times \Sigma _{2}$ solutions

Let us focus on $\,\text {AdS}_{2} \times \Sigma _{2}\,$ vacuum solutions with radii $\,L_{\text {AdS}_{2}}\,$ and $\,L_{\Sigma _{2}}$. The corresponding space-time geometry is specified by a metric of the form

45 $$\begin{aligned} d s^2 = - \frac{r^2}{L^2_{\text {AdS}_{2}}} \, d t^2 + \frac{L^2_{\text {AdS}_{2}}}{r^2} \, d r^2 + L_{\Sigma _{2}}^{2} \, d\Omega _{\Sigma _2}. \end{aligned}$$

The metric in (45) also describes the horizon of a static and extremal black hole, like the one in (1), (2), so these solutions are sometimes referred to as black hole horizon solutions.

An ansatz for the fields in the vector–tensor sector of the Lagrangian (4) that is compatible with the space-time symmetries takes the form [26] (see [35] for a tensor gauge-equivalent choice):

46 $$\begin{aligned} \mathcal {A}^\Lambda= & {} \mathcal {A}_t{}^\Lambda (r) \, d t - p^\Lambda \, \frac{ \cos \sqrt{\kappa } \, \theta }{ \kappa } \, d \phi , \nonumber \\ \tilde{\mathcal {A}}_\Lambda= & {} \tilde{\mathcal {A}}_t{}_\Lambda (r) \, d t - e_\Lambda \, \frac{ \cos \sqrt{\kappa } \, \theta }{ \kappa } \, d \phi , \nonumber \\ \mathcal {B}_{\alpha }= & {} b_{\alpha }(r) \, \frac{ \sin \sqrt{\kappa } \, \theta }{ \sqrt{\kappa } } \, d \theta \wedge d \phi . \end{aligned}$$

As a consequence of (11) and (46), this sector of the theory is encoded in a vector of charges $\,\mathcal {Q}\,$ of the form $\,\mathcal {Q}^{M} = \left( \mathfrak {p}^{\Lambda }, \mathfrak {e}_{\Lambda } \right) ^{T}\,$ with

47 $$\begin{aligned} \mathfrak {p}^{\Lambda }=p^{\Lambda } - \frac{1}{2} \, \Theta ^{\Lambda \, \alpha } \, b_{\alpha } \quad \text { and }\quad \mathfrak {e}_{\Lambda }=e_{\Lambda } + \frac{1}{2} \, \Theta _{\Lambda }{}^{\alpha } \, b_{\alpha }, \end{aligned}$$

which generically depends on vector charges $\,(p^{\Lambda },e_{\Lambda })\,$ as well as on the $\,\theta$-$\varphi \,$ components $\,b_{\alpha }\,$ of the tensor fields in (46). The latter play a role only in the massive IIA models where the gaugings are of dyonic type.

In the presence of hypermultiplets, quarter-BPS black hole horizon solutions with constant scalars require the set of algebraic equations [35] (see also [48])

48 $$\begin{aligned} \begin{aligned} \mathcal {Q}&= \kappa \, L_{\Sigma _{2}}^{2} \, \Omega \, \mathcal {M} \, \mathcal {Q} ^{x} \, \mathcal {P}^{x} - 4 \, \text {Im}(\bar{\mathcal {Z}} \, \mathcal {V}), \\ \dfrac{L_{\Sigma _{2}}^{2}}{L_{\text {AdS}_{2}}}&= -2 \, \mathcal {Z} \, e^{-i \beta }, \\ \left\langle \mathcal {K}^{u}, \mathcal {V} \right\rangle&= 0, \end{aligned} \end{aligned}$$

defined in terms of a central charge $\,\mathcal {Z}(z^{i})=\left\langle \mathcal {Q}, \mathcal {V} \right\rangle \,$, the scalar matrix $\,\mathcal {M}(z^{i})\,$ in (10) and $\,\mathcal {Q}^{x} = \left\langle \mathcal {P}^{x}, \mathcal {Q} \right\rangle$. The phase $\,\beta \,$ is associated with the complex function

49 $$\begin{aligned} W=e^{U} (\mathcal {Z} + i \, \kappa L_{\Sigma _{2}}^2 \, \mathcal {L})= |W| \, e^{i \beta }, \end{aligned}$$

which depends on the central charge $\,\mathcal {Z}(z^i)\,$ and a superpotential $\,\mathcal {L}(z^{i},q^{u})=\left\langle \mathcal {Q}^{x} \mathcal {P}^{x}, \mathcal {V} \right\rangle$.

With the aim of constructing later on asymptotically $\hbox {AdS}_{4}$ black holes of the universal type in (1), (2), we are concentrating on the configurations of the $\,z^{i}\,$ scalars found in the previous section to be compatible with $\hbox {AdS}_{4}$ solutions preserving $\,\mathcal {N}=2\,$ supersymmetry.

#### M-theory

As for the case of $\hbox {AdS}_{4}$ solutions, the third condition in (48) automatically discards $\,\text {AdS}_{2} \times \Sigma _{2}\,$ solutions in the $\,\text {SO}(7,1)\,$ and $\,\text {SO}(5,2)\,$ theories. Nevertheless, black hole horizon solutions with non-zero magnetic charges $\,p^{\Lambda }\,$ exist in all the remaining $\,\text {SO}(p,q)\,$ theories.

In the case of a trivial configuration of the universal hypermultiplet, various solutions are found for the $\,\text {SO}(8)\,$, $\,\text {SO}(4,4)\,$ and $\,\text {SO}(6,2)_{a,b}\,$ theories. Of special interest will be those of the $\,\text {SO}(8)\,$ and $\,\text {SO}(4,4)\,$ theories with the scalars $\,z^{i}\,$ being fixed at the values in (30) and (31), respectively. These solutions are supported by charges of the form

50 $$\begin{aligned}&\text {SO(8)} : p^{0}=-p^{1}=-p^{2}=-p^{3}=\tfrac{1}{4 g}, \end{aligned}$$

51 $$\begin{aligned}&\text {SO(4,4)} : p^{0}=p^{1}=p^{2}=-p^{3} = - \tfrac{1}{4 g}, \end{aligned}$$

and both have a hyperbolic horizon $\,\Sigma _{2}=\mathbb {H}^2\,$ ($\kappa = - 1$), a phase $\,\beta =0\,$ in (49) and radii given by

52 $$\begin{aligned} L^2_{\text {AdS}_{2}} = \tfrac{1}{8} \, g^{-2}, \quad L^2_{\mathbb {H}^{2}} = \tfrac{1}{4} \, g^{-2}. \end{aligned}$$

For the SO(6,2)$_{a}$ theory there are solutions with the scalars $\,z^{i}\,$ being also fixed as in (30) and (31). They are respectively supported by charges of the form

53 $$\begin{aligned}&\text {SO(6,2)}_{a} : p^{0}=\tfrac{1}{g}, \quad p^{1}=p^{2}=p^{3}=0, \end{aligned}$$

54 $$\begin{aligned}&\text {SO(6,2)}_{a} : p^{0}=p^{1}=p^{2}=0, \quad p^{3}=\tfrac{1}{g}, \end{aligned}$$

and both have a spherical horizon $\,\Sigma _{2}=\mathbb {S}^2\,$ ($\kappa = 1$), a phase $\,\beta =0\,$ in (49) and radii given by

55 $$\begin{aligned} L^2_{\text {AdS}_{2}} = \tfrac{1}{2} \, g^{-2}, \quad L^2_{\mathbb {S}^2} = \tfrac{1}{2} \, g^{-2}. \end{aligned}$$

Analogue solutions can also be found in the SO(6,2)$_{b}$ theory.

In the case of requiring a non-trivial configuration of the universal hypermultiplet, only the $\,\text {SO}(8)\,$ and $\,\text {SO}(4,4)\,$ theories turn out to accommodate black hole horizon solutions. These have the scalars $\,z^{i}\,$ fixed as in (35) and (36) and the universal hypermultiplet set as in (34). The solutions are supported by charges of the form

56 $$\begin{aligned}&\text {SO(8)} : p^{0} = -3p^{1} = -3p^{2} = -3p^{3} = \tfrac{1}{2 g}, \end{aligned}$$

57 $$\begin{aligned}&\text {SO(4,4)} : p^{0} = 3p^{1} = 3p^{2} = -3p^{3} = - \tfrac{1}{2 g}, \end{aligned}$$

and both have a hyperbolic horizon $\,{\Sigma _{2}=\mathbb {H}^2}\,$ (${\kappa =-1}$), a phase $\,\beta =0\,$ in (49) and radii given by

58 $$\begin{aligned} L^2_{\text {AdS}_{2}} = \tfrac{1}{6 \sqrt{3}} \, g^{-2}, \quad L^2_{\mathbb {H}^2} = \tfrac{1}{3 \sqrt{3}} \, g^{-2}. \end{aligned}$$

Lastly, additional $\,\text {AdS}_{2} \times \Sigma _{2}\,$ solutions equivalent to the ones presented above are obtained upon replacing $\,\mathcal {Q} \rightarrow -\mathcal {Q}\,$ and $\,\beta \rightarrow \beta + \pi$.

#### Massive IIA

**Table 2:** Vector charges supporting supersymmetric $\,\text {AdS}_{2} \times \Sigma _{2}\,$ solutions in the massive IIA models

$\,\, \mathcal {Q} \,\,$	$\,\,\text {ISO}(7)\,\,$	$\,\,\text {ISO}(6,1)\,\,$	$\,\,\text {ISO}(5,2)\,\,$	$\,\,\text {ISO}(4,3)\,\,$
$m^{-2/3} \, g^{5/3} \, \mathfrak {p}^0$	$\phantom {-}\frac{1}{6}$	$\phantom {-}\frac{1}{14}$	$-\frac{1}{14}$	$-\frac{1}{6}$
$g \, p^1$	$-\frac{1}{3}$	$-\frac{1}{7}$	$-\frac{1}{7}$	$-\frac{1}{3}$
$g \, p^2$	$-\frac{1}{3}$	$-\frac{1}{7}$	$-\frac{1}{7}$	$-\frac{1}{3}$
$g \, p^3$	$-\frac{1}{3}$	$-\frac{5}{7}$	$\phantom {-}\frac{5}{7}$	$\phantom {-}\frac{1}{3}$
$m^{1/3} \, g^{2/3} \, \mathfrak {e}_0$	$-\frac{1}{6}$	$\phantom {-}\frac{1}{14}$	$\phantom {-}\frac{1}{14}$	$-\frac{1}{6}$
$m^{-1/3} \, g^{4/3} \, e_1$	$-\frac{1}{6}$	$-\frac{3}{14}$	$\phantom {-}\frac{3}{14}$	$\phantom {-}\frac{1}{6}$
$m^{-1/3} \, g^{4/3} \, e_2$	$-\frac{1}{6}$	$-\frac{3}{14}$	$\phantom {-}\frac{3}{14}$	$\phantom {-}\frac{1}{6}$
$m^{-1/3} \, g^{4/3} \, e_3$	$-\frac{1}{6}$	$-\frac{3}{14}$	$-\frac{3}{14}$	$-\frac{1}{6}$
$\beta$	$\frac{\pi }{6}$	$-\frac{\pi }{6}$	$-\frac{5\pi }{6}$	$\frac{5\pi }{6}$

Quarter-BPS $\,\text {AdS}_{2} \times \Sigma _{2}\,$ solutions exist in all the $\,\text {ISO}(p,q)\,$ theories for different values of the vector of charges $\,\mathcal {Q}\,$ (see Table 2). The solutions have non-zero magnetic $\,\mathfrak {p}^{\Lambda }\,$ and electric $\,\mathfrak {e}_{\Lambda }\,$ charges in (47), and require $\,|\mathbf {\zeta }|^2=0\,$ and a non-trivial value of the dilaton $\,e^{\phi }\,$ in the universal hypermultiplet.

For the $\,\text {ISO}(7)\,$ and $\,\text {ISO}(4,3)\,$ theories the scalars $\,z^{i}\,$ are fixed at the values in (41) and (42), respectively, whereas the dilaton takes the value in (43). In both cases the solution has a hyperbolic horizon $\,{\Sigma _{2}=\mathbb {H}^2}\,$ ($\kappa =-1$) and radii given by

59 $$\begin{aligned} L^2_{\text {AdS}_{2}} = \frac{1}{4 \sqrt{3}} \, g^{-\frac{7}{3}}\, m^{\frac{1}{3}}, \quad L^2_{\mathbb {H}^2} = \frac{1}{2 \sqrt{3}} \, \, g^{-\frac{7}{3}}\, m^{\frac{1}{3}}. \end{aligned}$$

For the $\,\text {ISO}(5,2)\,$ and $\,\text {ISO}(6,1)\,$ theories the scalars $\,z^{i}\,$ are also fixed at the values in (41) and (42), respectively. However the dilaton in the universal hypermultiplet is fixed at the value

60 $$\begin{aligned} e^{\phi } = \frac{\sqrt{2}}{\sqrt{3}} \, \left( \frac{g}{m}\right) ^{\frac{1}{3}}. \end{aligned}$$

The solutions have a spherical horizon $\,\Sigma _{2}=\mathbb {S}^2\,$ (${\kappa =+1}$) and radii given by

61 $$\begin{aligned} L^2_{\text {AdS}_{2}} = \frac{3 \sqrt{3}}{4} \, g^{-\frac{7}{3}}\, m^{\frac{1}{3}}, \quad L^2_{\mathbb {S}^2} =\frac{3 \sqrt{3}}{14} \, \, g^{-\frac{7}{3}}\, m^{\frac{1}{3}}. \end{aligned}$$

Once again, a set of equivalent solutions is obtained upon replacing $\,\mathcal {Q} \rightarrow -\mathcal {Q}\,$ and $\,\beta \rightarrow \beta + \pi$.

### $\hbox {AdS}_{4}$ black hole solutions

Extremal R–N black holes interpolating between the (charged version [49] of) $\,\text {AdS}_{4}\,$ and $\,{\text {AdS}_{2} \times \Sigma _{2}}\,$ solutions previously found can be constructed. These black holes have constant scalars and can be viewed as non-minimal M-theory and massive IIA incarnations of the universal black hole in (1), (2) with a hyperbolic horizon.

In the context of M-theory reduced on $\,\text {H}^{(p,q)}\,$ spaces, two versions of such a black hole occur in each of the $\,\text {SO}(8)\,$ and $\,\text {SO}(4,4)\,$ theories. The first one involves a trivial configuration of the universal hypermultiplet and interpolates between an $\hbox {AdS}_{4}$ geometry with radius (32) in the ultraviolet (UV at $\,r \rightarrow \infty \,$) and an $\,\text {AdS}_{2} \times \mathbb {H}^{2}\,$ geometry with radii (52) in the infrared (IR at $\,r \rightarrow r_{h}\,$). The case of the SO(8) theory arising from $\,\text {H}^{(8,0)}=\text {S}^7\,$ has been extensively studied in the literature, see e.g. [2, 27, 50], also from a holographic perspective [1, 13–15]. The second version involves a non-trivial configuration of the universal hypermultiplet (34) and interpolates between an $\hbox {AdS}_{4}$ geometry with radius (37) in the UV and an $\,\text {AdS}_{2} \times \mathbb {H}^{2}\,$ geometry with radii (58) in the IR.^4^ For the SO(8) theory, it would be interesting to perform a holographic counting of $\hbox {AdS}_{4}$ black hole microstates in the field theory context of [47].

In the context of massive IIA reduced on $\,\text {H}^{(p,q)}\,$ spaces, there exists a universal black hole both in the $\,\text {ISO}(7)\,$ and $\,\text {ISO}(4,3)\,$ theories. It involves a non-trivial value of the dilaton field in the universal hypermultiplet (43) and interpolates between an $\hbox {AdS}_{4}$ geometry with radius (44) in the UV and an $\,\text {AdS}_{2} \times \mathbb {H}^{2}\,$ geometry with radii (59) in the IR. In the case of the ISO(7) theory arising from $\,\text {H}^{(7,0)}=\text {S}^6\,$, such a black hole has recently been constructed in [26, 28] and connected to a universal RG flow across dimensions in [1] (see [16, 17]) using holography.

## Summary and final remarks

In this note we have investigated supersymmetric $\,\text {AdS}_{4}\,$, $\,\text {AdS}_{2} \times \Sigma _{2}\,$ and universal $\hbox {AdS}_{4}$ black hole solutions in non-minimal $\,\mathcal {N}=2\,$ supergravity models with three vector multiplets (STU-model) and Abelian gaugings of the universal hypermultiplet. We have performed a systematic characterisation of $\,\mathcal {N}=2\,$ models that arise from 11D and massive IIA supergravity when reduced on $\,\text {H}^{(p,q)}\,$ spaces down to four dimension. More concretely, the models correspond to the $\,\text {U}(1)^2\,$ invariant sector of the $\,\text {SO}(p,q)\,$ (M-theory) and $\,\text {ISO}(p,q)\,$ (massive IIA) gauged maximal supergravities resulting upon reduction of the higher-dimensional theories. In M-theory models, the gaugings involve a duality-hidden symmetry pair of the universal hypermultiplet. In contrast, only duality symmetries of the universal hypermultiplet are gauged in massive IIA models. Supersymmetric solutions turn to populate different domains of the Kähler cone both in the M-theory and massive IIA cases.

Future lines to explore are immediately envisaged. The first one is the higher-dimensional description of the solutions based on non-compact $\,\text {H}^{(p,q)}\,$ reductions. Such solutions often lie in a different domain of the Kähler cone than their counterparts in sphere reductions. In addition, for the case of sphere reductions, it would also be interesting to have a higher-dimensional picture of the $\hbox {AdS}_{4}$ black holes involving a non-trivial universal hypermultiplet as the gravitational backreaction of bound states of M-/D-branes wrapping a Riemann surface [52, 53]. To this end, connecting the four-dimensional fields and charges to higher-dimensional backgrounds of 11D and massive IIA supergravity is required. A suitable framework to obtain such uplifts is provided by the duality-covariant formulation of 11D [54] and massive IIA supergravity [55] in terms of an exceptional field theory. Using this framework, general uplifting formulae have been systematically derived for the consistent truncation of M-theory and massive IIA on spheres^5^ and hyperboloids [32, 57] down to a gauged maximal supergravity in four dimensions. It would then be interesting to uplift the $\mathcal {N}=2$ supergravity models constructed in this note, and therefore any of their solutions, to backgrounds of 11D and massive IIA supergravity on $\text {H}^{(p,q)}$.

Lastly the $\mathcal {N}=2$ formulation of M-theory models presented in this note can be straightforwardly used to describe $\,\text {SO}(p,q)\,$ models with dyonic gaugings, the prototypical example being the $\omega$-deformed version of the $\,\text {SO}(8)\,$ theory [58]. In this theory, and unlike for the original STU-model with only vector multiplets [59], the presence of the universal hypermultiplet makes the Lagrangian in (4) sensitive to the electric-magnetic deformation parameter $\,\omega \,$ and affects the structure of supersymmetric solutions. For instance, two inequivalent $\,\mathcal {N} = 2\,$ $\hbox {AdS}_{4}$ solutions preserving an $\,\text {SU}(3) \times \text {U}(1)\,$ symmetry in the full theory appear at generic values of $\,\omega \,$ [60]. It is therefore interesting to understand the structure of $\hbox {AdS}_{4}$ black hole solutions in this setup [61] for which a higher-dimensional description remains elusive [62]. We hope to come back to these and related issues in the near future.

## Appendix: Embedding in maximal supergravity

In this appendix we provide the embedding of the non-minimal $\,\mathcal {N}=2\,$ supergravity models analysed in this note into $\,\mathcal {N}=8\,$ (maximal) supergravity [63]. In its ungauged version, maximal supergravity possesses an ${\text {E}}_{7(7)}$ global symmetry group. This group plays a central role in systematically constructing the gauged versions of the theory using the embedding tensor formalism [64]: in a gauged maximal supergravity, a specific subgroup of ${\text {E}}_{7(7)}$ is promoted from global to local in what is known as the gauging procedure. In this note we focus on $\,\text {SO}(p,q)\,$ and $\,\text {ISO}(p,q)\,$ subgroups of $\, \text {SL}(8) \subset \text {E}_{7(7)}$. These gauged supergravities appear upon reduction of 11D and massive IIA supergravity on $\,\text {H}^{(p,q)}\,$ spaces.

Let us start by introducing a fundamental SL(8) index $\,A=1,...8$. In the SL(8) basis, the ${\text {E}}_{7(7)}$ generators $\,t_{\alpha =1,...,133}\,$ have a decomposition $\,{\mathbf {133}} \rightarrow {\mathbf {63}} + {\mathbf {70}}$. These are the $\,{\mathbf {63}}\,$ generators $\,{t_{A}}^{B}\,$ of SL(8), with vanishing trace $\,{t_{A}}^{A}=0\,$, together with $\,{\mathbf {70}}\,$ generators $\,t_{ABCD}=t_{[ABCD]}$. The fundamental representation of ${\text {E}}_{7(7)}$ decomposes as $\,{\mathbf {56}} \rightarrow {\mathbf {28}} + {\mathbf {28'}}\,$, which translates into a splitting of the ${\text {E}}_{7(7)}$ fundamental index of the form $\,_\mathbb {M} \rightarrow _{[AB]} \oplus ^{[AB]}$. The entries of the $\,56 \times 56\,$ matrices $\,{[t_{\alpha }]_{\mathbb {M}}}^{\mathbb {N}\,}$ are given by

62 $$\begin{aligned} {[{t_{A}}^{B}]_{[CD]}}^{[EF]}= & {} 4 \, \left( \delta _{[C}^{B} \, \delta _{D]A}^{EF} + \frac{1}{8} \, \delta _{A}^{B} \, \delta _{CD}^{EF} \right) ,\nonumber \\ {[{t_{A}}^{B}]^{[EF]}}_{[CD]}= & {} - {[{t_{A}}^{B}]_{[CD]}}^{[EF]} , \end{aligned}$$

for the SL(8) generators $\,{t_{A}}^{B}$. The generators $\,t_{ABCD}\,$ completing SL(8) to ${\text {E}}_{7(7)}$ take the form

63 $$\begin{aligned} {[}t_{ABCD}]_{[EF][GH]}= & {} \frac{2}{4!} \, \epsilon _{ABCDEFGH} , \nonumber \\ {[}t_{ABCD}]^{[EF][GH]}= & {} 2 \, \delta _{ABCD}^{EFGH} . \end{aligned}$$

The electric $\,\text {SO}(p,q)\,$ and dyonic $\,\text {ISO}(p,q)\,$ gaugings of maximal supergravity belong to $\,\text {SL}(8)\subset \text {E}_{7(7)}\,$ and are specified by an embedding tensor [64] of the form

64 $$\begin{aligned} \Theta _{AB}{}^{C}{}_{D} = 2 \, \delta _{[A}^{C} \, \eta _{B]D} , \quad \Theta ^{AB}{}^{C}{}_{D} = 2 \, \delta ^{[A}_{D} \, \tilde{\eta }^{B]C}. \end{aligned}$$

The matrices $\,\eta _{AB}\,$ and $\,\tilde{\eta }^{AB}\,$ associated with the different gaugings are collected in Table 3.

**Table 3:** Matrices $\,\eta _{AB}\,$ and $\,\tilde{\eta }^{AB}\,$ specifying the $\,\text {SO}(p,q)\,$ and $\,\text {ISO}(p,q)\,$ gaugings of maximal supergravity

gauging	$\,\,\eta _{AB}\,\,$	$\,\,\tilde{\eta }^{AB}\,\,$
$\text {SO}(8)$	$\text {diag}(+1,\mathbb {I}_{2},\mathbb {I}_{2},\mathbb {I}_{2},+1)$	0
$\text {SO}(7,1)$	$\text {diag}(+1,\mathbb {I}_{2},\mathbb {I}_{2},\mathbb {I}_{2},-1)$	0
$\text {SO}(6,2)_{a}$	$\text {diag}(-1,\mathbb {I}_{2},\mathbb {I}_{2},\mathbb {I}_{2},-1)$	0
$\text {SO}(6,2)_{b}$	$\text {diag}(+1,\mathbb {I}_{2},\mathbb {I}_{2},-\mathbb {I}_{2},+1)\,\,$	0
$\text {SO}(5,3)$	$\text {diag}(+1,\mathbb {I}_{2},\mathbb {I}_{2},-\mathbb {I}_{2},-1)$	0
$\text {SO}(4,4)$	$\text {diag}(-1,\mathbb {I}_{2},\mathbb {I}_{2},-\mathbb {I}_{2},-1)$	0
$\text {ISO}(7)$	$\text {diag}(+1,\mathbb {I}_{2},\mathbb {I}_{2},\mathbb {I}_{2},0)$	$\text {diag}(0_{7},-m)\,\,$
$\text {ISO}(6,1)$	$\text {diag}(-1,\mathbb {I}_{2},\mathbb {I}_{2},\mathbb {I}_{2},0)$	$\text {diag}(0_{7},-m)\,\,$
$\text {ISO}(5,2)$	$\text {diag}(+1,\mathbb {I}_{2},\mathbb {I}_{2},-\mathbb {I}_{2},0)$	$\text {diag}(0_{7},-m)\,\,$
$\text {ISO}(4,3)$	$\text {diag}(-1,\mathbb {I}_{2},\mathbb {I}_{2},-\mathbb {I}_{2},0)$	$\text {diag}(0_{7},-m)\,\,$

The scalar sector of maximal supergravity consists of $\,70\,$ fields spanning a coset space $\,\text {E}_{7(7)}/\text {SU}(8)$. However, in this note we concentrate on non-minimal $\,\mathcal {N}=2\,$ supergravity models associated with the $\,\text {U}(1)^2\,$ invariant sector of the theory. The scalars in this sector span an $\,[\text {SL}(2)/\text {SO}(2)]^3 \, \times \, \text {SU}(2,1)/(\text {SU}(2)\times \text {U}(1))\,$ coset space associated with the following ${\text {E}}_{7(7)}$ generators. The $\,[\text {SL}(2)/\text {SO}(2)]^3\,$ factor is associated with

65 $$\begin{aligned} H_{\varphi _1}= & {} {t_{4}}^{4} + {t_{5}}^{5} + {t_{6}}^{6} + {t_{7}}^{7} - {t_{1}}^{1} - {t_{8}}^{8} - {t_{2}}^{2} - {t_{3}}^{3} , \nonumber \\ H_{\varphi _2}= & {} {t_{2}}^{2} + {t_{3}}^{3} + {t_{6}}^{6} + {t_{7}}^{7} - {t_{1}}^{1} - {t_{8}}^{8} - {t_{4}}^{4} - {t_{5}}^{5} , \nonumber \\ H_{\varphi _3}= & {} {t_{2}}^{2} + {t_{3}}^{3} + {t_{4}}^{4} + {t_{5}}^{5} - {t_{1}}^{1} - {t_{8}}^{8} - {t_{6}}^{6} - {t_{7}}^{7} , \nonumber \\ g_{\chi _1}= & {} t_{1238},g_{\chi _2} = t_{1458}, g_{\chi _3} = t_{1678} . \end{aligned}$$

The $\,\text {SU}(2,1)/(\text {SU}(2)\times \text {U}(1))\,$ factor is associated with

66 $$\begin{aligned} H_{\phi }= & {} \tfrac{1}{2} \, ({t_{8}}^{8}-{t_{1}}^{1}), g_{\sigma } \,\,\, = \,\,\, {t_{8}}^{1} , \nonumber \\ g_{\zeta }= & {} t_{8357} - t_{8346} - t_{8256} - t_{8247} , \nonumber \\ g_{\tilde{\zeta }}= & {} t_{8246} - t_{8257} - t_{8347} - t_{8356}. \end{aligned}$$

Using the above generators, the coset representative $\mathcal {V}=\mathcal {V}_{\text {SK}} \times \mathcal {V}_{\text {QK}}$ is obtained upon the exponentiations

67 $$\begin{aligned} \mathcal {V}_{\text {SK}}= & {} \prod _{i} e^{-12 \, \chi _{i} \, g_{\chi _{i}}} \,\,\, e^{\frac{1}{4} \, \varphi _{i} \, H_{\varphi _{i}}} , \nonumber \\ \mathcal {V}_{\text {QK}}= & {} e^{\sigma \, g_{\sigma }} \,\,\, e^{-6 \,(\zeta \, g_{\zeta } \,+\, \tilde{\zeta } \, g_{\tilde{\zeta }}) } \,\,\, e^{-2 \, \phi \, H_{\phi }}. \end{aligned}$$

Starting from the representative $\,\mathcal {V}\in \text {E}_{7(7)}/\text {SU}(8)\,$, the scalar matrix $\,\mathcal {M}_{\mathbb {MN}}\,$ entering the Lagrangian of $\,\mathcal {N}=8\,$ supergravity [64] is obtained as $\,{\mathcal {M}=\mathcal {V} \, \mathcal {V}^{t}}$. Plugging this matrix $\,\mathcal {M}\,$ into the kinetic terms of the maximal theory, $\,{e^{-1} \mathcal {L}_{\text {kin}}=\frac{1}{96} \text {Tr} [ D_{\mu } \mathcal {M} \, D^{\mu }\mathcal {M}^{-1}]}\,$, we obtain the kinetic terms for the $\,\mathcal {N}=2\,$ models in (5) and (12). The scalar potential and covariant derivatives of the $\,\mathcal {N}=2\,$ models can be obtained from the ones of the maximal theory upon identifying the electric vectors $\,{\mathcal {A}^{AB}=\mathcal {A}^{[AB]}}\,$ in the maximal theory as $\,\{\mathcal {A}^0,\mathcal {A}^1,\mathcal {A}^2,\mathcal {A}^3 \} \equiv \{ \mathcal {A}^{18}, \mathcal {A}^{23}, \mathcal {A}^{45}, \mathcal {A}^{67} \}\,$ (and similarly for the magnetic vectors $\,{\tilde{\mathcal {A}}_{AB}}\,$), and then truncating away the non-singlet fields. We have verified that the $\,\mathcal {N}=8\,$ results precisely match the $\,\mathcal {N}=2\,$ results obtained from (14) and (22).

## Footnotes

1 See [5] for a generalisation to asymptotically AdS black branes in various dimensions.

2 Einstein-scalar systems associated with $\,{\mathbb {Z}_{2} \times \text {SO(3)}}\,$ and $\,\text {SU}(3)\,$ invariant sectors of such $\,\text {H}^{(p,q)}\,$ reductions were presented in [30].

3 Note that $z^{i}_{\text {here}} = e^{i \frac{\pi }{3}} z_{[10]}^{i}$ as a consequence of the sign choice $m_{\text {here}}=-m_{[10]}$.

4 See [48] for a similar model based on the $\,Q^{111}\,$ truncation of M-theory [51].

5 See [56] for a recent derivation of the complete $\,D=11\,$ embedding of $\,\text {SO}(8)\,$ gauged maximal supergravity using the method introduced in [22] for the $\,D=10\,$ embedding of the $\,\text {ISO}(7)\,$ theory.
